# The influence of heavy metals on the shape and asymmetry of wings of female *Polistes nimpha* (Hymenoptera, Vespidae) living on contaminated sites

**DOI:** 10.1007/s10646-021-02449-8

**Published:** 2021-07-14

**Authors:** Anna Mielczarek, Łukasz Mielczarek, Elżbieta Wojciechowicz-Żytko

**Affiliations:** 1 University of Agriculture in Krakow, Faculty of Biotechnology and Horticulture, Department of Biology, Physiology and Plant Protection, Al. 29 Listopada 54, 31-425 Krakow, Poland; 2 Krakow Municipal Greenspace Authority, Reymonta 20, 30-059 Krakow, Poland

**Keywords:** Heavy metals, Fluctuating asymmetry, *Polistes nimpha*, Wings, Pollution

## Abstract

The aim of the present study was to determine the fluctuating asymmetry of the first pair of wings in females *Polistes nimpha* (Christ, 1791) living in an environment contaminated with heavy metals. The average concentration of Zn, Cd and Pb in the bodies of the insects varied depending on the distance from the source of contamination, reaching the highest values on the site closest to the source of contamination and the lowest at the most distant site. As a result of the morphometric analyses, significant differences were found in the asymmetry values of the first pair of wings depending on the level of Zn, Cd, Pb accumulated by the wasps. In the case of shape asymmetry, differences were found for all the effects studied (year of capture and site). Significant differences were also found in the size of wings between individuals captured on Sites 1 and 2 and those caught on Site 3. Specimens caught on site characterized by the lowest concentration of heavy metals in the topsoil, proved to be significantly larger than the insects collected on the other sites. There were no differences in the size of individuals between the different years of capture. Based on the results obtained by us, it can be assumed that the wings of *P. nimpha* females may become a useful object in studying the impact of environmental stress of Zn, Cd and Pb pollution on the symmetry of their wings.

## Introduction

Heavy metals are widely distributed in the environment through natural sources (e.g. bedrock weathering), but more and more attention is now being paid to this group of metals as they are inextricably linked with everyday human activities (mining, processing and automotive industries, agriculture, etc.) (Alloway 2013).

Despite the fact that some metals are used by living organisms and are necessary for their proper development, they become toxic to them after exceeding critical levels. The remaining metals are toxic regardless of the amount absorbed by living organisms (Boyd and Rajakaruna 2013). Too high a concentration of both these groups of metals in the habitat of living organisms may constitute for them a strong stress factor determining their development. For these reasons, heavy metals have been widely studied in terms of their negative effects on the human body (Liu et al. 2013; Qing et al. 2015), but research is also conducted on other living organisms, such as insects. Evaluation of the impact of heavy metals on insects is based on determining, *inter alia*, the influence of these elements on the reproductive capacity and proliferation in contaminated areas (Moroń et al. 2013), the extent of parasitization, species diversity (Szentgyörgyi et al. 2011), etc.

One of the tools also used to estimate the influence of heavy metals on living organisms is analysis of fluctuating asymmetry in individual specimens. In theory, any organism with a bilateral structure is characterized by the presence of perfect bilateral symmetry (a normally distributed pattern of symmetry in a population) (Freeman et al. 1993). In reality, however, small, randomly occurring deviations from the symmetry of bilateral features are widespread in nature, resulting from, for example, low developmental stability of the organism. They are called fluctuating asymmetry. An organism that develops under unfavourable exo- or endogenous conditions is often characterized by a greater degree of asymmetry (Daloso 2014; Palmer and Strobeck 1986; Van Valen 1962).

Research on fluctuating asymmetry in response to a variety of stresses has been conducted with a variety of organisms: plants (Alves-Silva and Del-Claro 2013; Ivanov et al. 2015), birds (Herring et al. 2016; Minias et al. 2013), fish (Özsoy et al. 2007; Tocts et al. 2016), mammals (Cánovas et al. 2015; Sánchez-Chardi et al. 2013). They have also concerned various types of stress factors, e.g. temperature (Bjorksten et al. 2001; Chang et al. 2007), population density (Gibbs and Breuker 2006; Mpho et al. 2000), the extent of parasitization (Ward et al. 1998), exposure to contamination with pesticides (Abaga et al. 2011; Hardersen et al. 1999) and heavy metals (Graham et al. 1993; Polak et al. 2004).

The aim of the present study was to determine the fluctuating asymmetry (FA) of the first pair of wings of females of the predatory species Polistes *nimpha* (Christ, 1791) living in an environment contaminated with heavy metals (Zn, Cd and Pb).

The results presented in this article are an extension of part of the research by Mielczarek and Wojciechowicz-Żytko (2020).

## Materials and methods

### Study sites

The study was carried out in the vicinity of Zakłady Górniczo-Hutnicze (ZGH) “Bolesław”––a mining and processing complex located in Bukowno near Olkusz (southern Poland) (50°30′28″N, 19°28′17″E). These plants, operating since 1967, are engaged in mining and processing of zinc and lead ores. Their operations contribute to environmental contamination with heavy metals such as Zn, Cd and Pd.

Based on the concentrations of heavy metals in the topsoil, which had been reported in the works by Grześ (2009) and Szentgyörgyi et al. (2011), and on personal observations, three sites were designated for the study, differing in terms of heavy metal concentrations in the soil and the distance from the source of contamination (0.44 km, 1.5 km, and 19.62 km, respectively). All three sites were warm, sunlit grasslands surrounded by Scots pine (*Pinus sylvestris* L.) trees with an admixture of other pioneering types of trees and shrubs (e.g. *Betula*, *Larix*, *Prunus*). Among the plants flourishing on the grasslands in great numbers were plants of the family Apiaceae (including *Pimpinella saxifraga* and *Daucus carota* L.), which were used by *P. nimpha* for hunting the prey.

In 2015, samples of topsoil at depths up to 20 cm were collected from all three sites and analyzed for Zn, Cd and Pb content. The samples were taken at random from an area of 1 km^2^, giving special consideration to the nesting sites of *P. nimpha* and their feeding grounds. The top layer of soil was sampled with a metal spatula, discarding the part of soil that came in contact with it (to eliminate the risk of sample contamination). Each sample was placed in a separate bag and transported to the laboratory. The samples were dried in the open air, milled and sieved through 0.2 mm sieves.

Forty topsoil samples were collected from each of the three sites; they were mixed to obtain 3 bulk (pooled) samples to represent each site in further analyses. The bulk soil samples were used to determine their granulometric composition by the Casagrande method, as modified by Prószyński, and soil pH using the potentiometric method in a 1:2 water:soil solution.

To determine the concentrations of heavy metals (Zn, Cd, Pb), the soil samples were dried, milled and homogenized. Weighed amounts of 0.5 g were each transferred to a vessel into which 10 ml of aqua regia was added, and mineralized. The resulting solutions were filtered, transferred to 50 ml flasks and rinsed with deionized water. Metal content was determined using the ICP-OES method (optical emission spectrometry with inductively coupled plasma – as recommended for the determination of metals in soil).

### Insects studied

The study was concerned with females of the predatory species *P. nimpha* (Christ, 1791). *P. nimpha* like other species of the genus Polistes is a social vespid wasp known to build relatively small paper nests which also reflect the English name of these insects – paper wasps. The species is abundant throughout Poland, living in warm, sunny places, where they build their nests. Numerous individuals of wasps develop in these nests. Their building of nests means that they are insects that persist in a given environment, which, combined with relatively poor flying ability, makes them ideal as bioindicators of the state of the environment (Hunt 2007; Prezoto and Gobbi 2005; Suzuki 1978).

Although flying close to the nest, females of the Polistes as predators penetrate its territory very actively in order to feed its larvae. The Polistes are known to feed its larvae mainly on caterpillars (Sumner and Cini 2021) which eat various plants so that it is expected that they have plenty of opportunity to be exposed to pollutants and accumulate a substantial amount during its relatively long life.

Imagines of the genus Polistes were caught in 2015–2017, in late July and early August, when they reach the highest numbers and still remain close to their nests. The “on sight” catching of live individuals was carried out with an entomological net, on warm days conducive to active foraging of adults. The insects were caught during active flight or while feeding on plants. The specimens were kept in separate Eppendorf vials with air access. The caught individuals were transported to the laboratory, where they were kept individually in boxes with a perforated lid at 25 °C for 48 h. The wasps were not fed to make them empty their intestinal contents; they were provided only with sterile swabs soaked in distilled water. The insects were then sacrificed by freezing, segregated by sex and recognized to the species on the basis of the morphological features contained in the Dvořák and Roberts (2006) entomological key, which allowed them to be easily and quickly distinguished from other Polistes species (antennae distinctly darkened above, last sternum black, clypeus with transverse black strip).

Females of the species *P. nimpha*, as the most abundant species, were selected for further analyses. The selected individuals were de-winged. The wings were placed in photo slides and scanned (at a resolution of 2400 dpi) with a Nikon Coolscan 5000 ED scanner, saving the image obtained in this way. Later, the wings were put together with the corresponding specimen, which was rinsed in distilled water (to remove remnants of impurities that could overestimate the results of analyses), and dried in laboratory conditions at a temperature of 20 °C.

The bodies of the insects were analyzed for heavy metals (Zn, Cd, Pb); the samples were mineralized by wet digestion in a semi-open system with heating plates (quartz glass). After digestion, all sub-samples, blanks and reference materials were flooded with 2 ml of acidic water (0.2% nitric acid). The solutions produced in this way were then used to determine the concentration of heavy metals (Cd, Pb and Zn) in them using atomic absorption spectrophotometers: PerkinELmer PinAAcle 900Z (Pb, Cd) in a graphite cuvette and PerkinElmer AAnalyst 200 (Zn) in an acetylene-air flame. The following wavelengths were used for the individual elements: Cd – 228.8 nm, Pb – 283.3 nm, Zn – 213.9 nm.

Photographs of the wings of the tested individuals were assigned to the appropriate side of the body. The image obtained by scanning the wings was prepared for further analyses using the IdentiFly software (Przybyłowicz et al. 2016); http://drawwing.org/identifly. Using the IdentiFly programme, 17 points of intersection of the veins were marked on the obtained image of the first pair of wings which are much larger than the hindwings and form the main flight surface, so that they are a good focus for comparisons of biometric characteristics like size and shape. Moreover it is known that wing size is strongly correlated with body size and has been used as an measure of body size in insects living in natural (Bullock 1999) and polluted areas (Moroń et al. 2013, Szentgyörgyi et al. 2017). Szentgyörgyi et al. (2017) report the correlation of body mass and wing size of mason bee in areas polluted by heavy metals.We expected that similar correlation of wing size, body size and body mass should occur also in individuals of Polistes.

No points were measured in the apical part of the wing because it had been damaged in many specimens over the course of their lifetime, and this would have reduced the size of the available test sample. Specimens whose wings were badly damaged were not subjected to morphometric analyses. Estimation of the shape based on geometric morphometrics requires relatively large sample sizes (Cardini and Elton 2007). It is recommended that, in multivariate analysis, the sample size of each group should markedly exceed the number of variables (Arnold 1983).

The coordinates of the landmarks were superimposed using full Procrustes fit in MorphoJ software (Klingenberg 2011). To calculate asymmetry Procrustes Anova (Klingenberg 2015) was calculated also using MorphoJ. The obtained data were subjected to MANOVA statistical tests. Wing size was analyzed as represented by the Centroid Size (Zelditch et al. 2004). Shape was described by Procrustes coordinates, which were scaled to the same size. Size asymmetry was measured as the absolute difference between the centroid sizes of the right and the left forewing divided by the mean centroid size. Shape asymmetry was measured as the Procrustes distance between the shapes of the right and the left wing, and it is further called Procrustes FA score. Centroid sizes and Procrustes FA scores were calculated with MorphoJ software (Klingenberg 2011).

## Results

### Soil

Each site was characterized by the presence of sandy soils, which were acidic or slightly acidic (pH 6.8, 6.02, and 5.59, respectively). The highest concentrations of heavy metals in the topsoil were recorded on Site 1, located in the immediate vicinity of ZGH “Bolesław”, where they were 4326.50 mg/kg Zn, 56.96 mg/kg Cd, and 3977.0 mg/kg Pb. The lowest concentrations of the analyzed elements were recorded on the site furthest away from the source of contamination (Site 3: 48.75 mg/kg Zn, 0.72 mg/kg Cd, and 25.43 mg/kg Pb) (Table 1.).

**Table 1:** Soil pH and Zn, Cd and Pb content in the topsoil of individual sites (Mielczarek and Wojciechowicz-Żytko 2020)

Site	Location	Elevation [m a.s.l.]	pH	Distance to the source of contamination [km]	Zn (mg/kg)	Cd (mg/kg)	Pb (mg/kg)
1	50°16′N 19°28′E	326	6.87	0.44	4326.50	56.96	3977.00
2	50°17′N 19°27′E	346	6.02	1.5	1856.30	35.21	915.88
3	50°26′N 19°35′E	412	5.59	19.63	48.75	0.72	25.43

### Insects

Individuals of the genus Polistes were collected in all three years of the study. They were classified into three species: *P. nimpha*, *P. dominula* and *P. biglumis* which are the only representatives of the genus in the studied area. The dominant species was *P. nimpha*, and that is why female specimens of this species were selected for further analyses––a total of 416 females of this species were caught.

The average concentration of heavy metals in the bodies of the insects varied depending on the distance from the source of contamination (Table 2), reaching the highest values on the site closest to the source of contamination (Site 1: 385.45 mg/kg Zn, 9.62 mg/kg Cd, and 6.61 mg/kg Pb), and the lowest at the most distant site (Site 3: 194.65 mg/kg Zn, 2.27 mg/kg Cd and 0.84 mg/kg Pb).

**Table 2:** Average concentration of Zn, Cd and Pb in the bodies of female *P. nimpha* wasps caught on different sites in years 2015–2017

Site	Zn (mg/kg)	Cd (mg/kg)	Pb (mg/kg)
1	385.45	9.62	6.61
2	258.24	2.43	2.04
3	194.65	2.27	0.84

A total of 268 pairs of wings were used in the morphometric analyses (Site 1 – 112, Site 2 – 74, Site 3 – 82). As a result of the analyses, significant differences were found in the asymmetry values of the first pair of wings depending on the level of heavy metals (Zn, Cd and Pb) accumulated by the wasps.

**Fig. 1:**
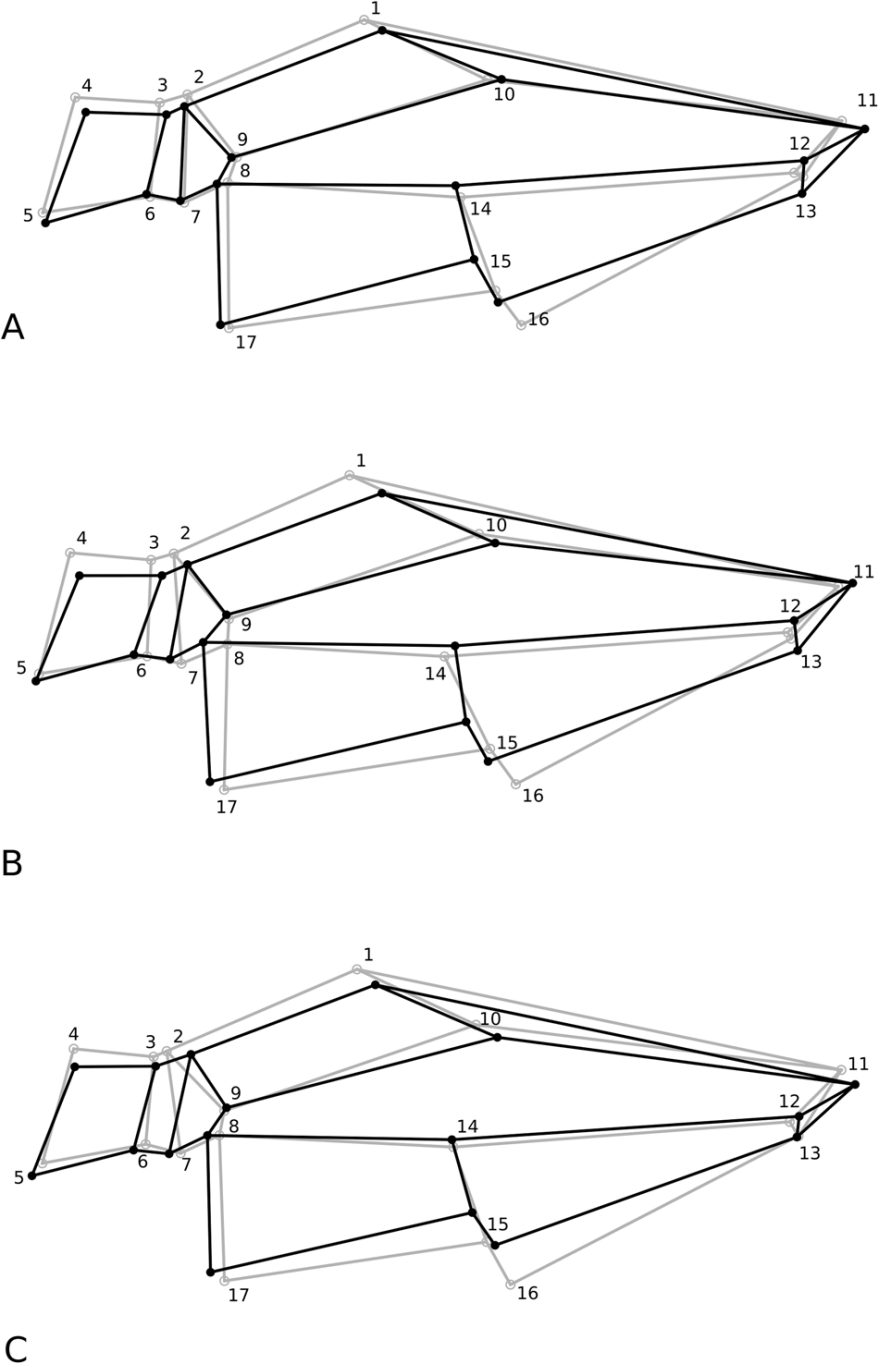
Diagram showing the differences in the shape of the veins between the right and left wings of *P. nimpha* females captured on Site 1 (**A**), 2 (**B**), and 3 (**C**) (Procrustes ANOVA). The differences are magnified 20× to make them more apparent. The black lines indicate the right wing, and the grey lines indicate the left wing

In the case of shape asymmetry, differences were found for all the effects studied (year of capture and site) (*p* = 0.001), with a significant difference between Site 1 and Site 3 (*p* = 0.01378) (Fig. 1).

Significant differences were also found in the size of wings between individuals captured on Sites 1 and 2 and those caught on Site 3 (*p* = 0.0004). Specimens caught on Site 3, the farthest away from the source of contamination and characterized by the lowest concentration of heavy metals in the topsoil, proved to be significantly larger than the insects collected on the other sites (Mann Whitney pairwise *p* = 0.0001455; *p* = 0.01527). There were no differences in the size of individuals between the different years of capture (*p* = 0.2684) (Table 3).

**Table 3:** Results of ANOVA: sums of squares (SS), mean squares (MS), degrees of freedom (df), *F* statistics and parametric *P*-values for each of the effects

Centroid size:					
Effect	SS	MS	*df*	*F*	*P* (param.)
Locality	307365.206020	153682.603010	2	8.04	0.0004
Year	50557.512899	25278.756449	2	1.32	0.2684
Individual	5029782.792646	19124.649402	263	317.94	<0.0001
Side	1026.884533	1026.884533	1	17.07	<0.0001
Ind*Side	16060.527544	60.151789	267		

Shape, Procrustes ANOVA:							
Effect	SS	MS	*df*	*F* (Goodall’s)	*P* (param.)	Pillai trace	*P* (param.)
Locality	0.00335033	0.0000558388	60	2.20	<0.0001	0.46	<0.0001
Year	0.00251390	0.0000418984	60	1.65	0.0013	0.42	<0.0001
Individual	0.20067318	0.0000254339	7890	9.55	<0.0001	24.47	<0.0001
Side	0.00172228	0.0000574092	30	21.56	<0.0001	0.59	<0.0001
Ind*Side	0.02132472	0.0000026623	8010				

## Discussion

Analysis of fluctuating asymmetry is recognized as one of the methods of assessing the impact of environmental stress on living organisms. Although not all the studies conducted so far confirm the occurrence of changes in the symmetry of individuals with respect to various stressors, other results clearly indicate the usefulness of FA analysis as a valid tool for studying environmental stress (Beasley et al. 2013). Hoffmann et al. (2005) state that any stress affecting insects during their life may change the shape of their wings in a specific way.

The aim of our study was to determine whether females of the predatory species *P. nimpha* would be characterized by increased levels of FA in response to elevated concentrations of heavy metals (Zn, Cd, and Pb) in their habitat. To verify our hypotheses, we had chosen to model their wings as a practically two-dimensional organ, characterized by the presence of characteristic points (intersections of the veins) to facilitate the analyses performed.

Insect wings have already been successfully used by other authors in this type of research (Benìtez et al. 2013 – *Diabrotica virgifera*, Costa et al. 2015 – *Drosophila antionetae*, Galbo and Tabugo 2014 – *Culex quinquefasciatus*, Nunes 2015, Quirog and Tabugo 2015 – *Aedes albopictus* Szentgyörgyi et al. 2016 – *Apis mellifera*).

As a result of our analyses, we found significant differences in the asymmetry values of the first pair of wings depending on the level of heavy metals (Zn, Cd and Pb) accumulated by wasps which are living in its natural but contaminated habitat.

A higher level of fluctuating asymmetry of various organs of the body in response to stress related to exposure to heavy metals has already been observed in aphids *Brevicoryne brassicae* (Görür 2006, 2009), *Chironomus* spp. (Al-Shami et al. 2010), dragonfly *Calopteryx maculata* (Kelliher 2004), and honeybee *Apis mellifera* (Abaga et al. 2011). On the other hand, there are reports in the literature that negate the occurrence of symmetry disorders caused by exposure to heavy metals, e.g. *Lasius flavus* (Grześ et al. 2015), *Formica pratensis* (Rabitsch 1997), *Chironomidae* spp. (Arambourou et al. 2012). When interpreting the cited studies, it should be remembered, however, that they were concerned with different taxa, and the analyses were performed on various external organs (wings, eyes, etc.).

In our study, we analyzed the size and shape of the wings of *P. nimpha* females captured along a concentration gradient. The results showed significant differences in the centroid size of the wings of individuals caught in the most contaminated areas (Site 1 and 2), compared to individuals collected on the site with the lowest degree of contamination (Site 3). In our study there were also no significant differences between years of capture. Similarly, Szentgyörgyi et al. (2017) had demonstrated significant differences in the size and shape of the wings of *Osmia bicornis* (Hymenoptera) captured on sites with different levels of contamination with heavy metals, but they did not show significant differences in the asymmetry of their wings depending on the year of capture.

Nijhout, Callier (2015); Nijhout, Grunert (2010) report that under stable environmental conditions the body size of a given species is relatively constant. However, it changes when a stress factor appears in the environment, such as temperature fluctuations or food availability. The wings forming during metamorphosis develop proportionally to the size of the entire insect. Therefore, based on the results obtained by us, we made an assumption that heavy metals (Zn, Cd and Pb) can be a stress factor affecting wing size of the adults of this species in its natural environment. This thesis is supported by the fact that there were no significant differences in the size of the wings depending on the year of capture, which theoretically excludes the impact of, for example, differences in atmospheric conditions between individual years that could have determined the development of larvae in Polistes colonies. However, this hypothesis would require laboratory tests to exclude the influence of other stress factors that could play a significant role in the individual development of wasps (e.g. food deficits, different calorific value of food consumed by the larvae). As this was a field experiment, we do not have data on the quantity and quality of food collected in each year of the study by individuals developing on the different sites.

A laboratory experiment on the effect of food availability on the size of insect wings had been conducted by Szentgyörgyi et al. (2016) and concerned representatives of the *Apis mellifera* species. In that study, only for drones were there significant differences in wing size between the families denied pollen and the control group. In the case of the worker caste, no similar relationship was found. However, the limited availability of food did not significantly change the size symmetry of the wings, regardless of sex.

Turillazzi (1980) reports that in the species *Polistes* *gallicus* there is a seasonal variation in body size; he recorded a gradual increase in the body size of females (queens and workers) in the summer. In the autumn, he did not observe statistically significant differences in size between the representatives of the two groups. Haggard and Gamboa (2012) also indicated that the body size of the species *Polistes metricus* varies during the season.

Taking into account the research results of the above-cited authors, the *P. nimpha* specimens captured by us were collected in the same period on all three sites to exclude the possibility of a similar phenomenon of different size of *P. nimpha* specimens, which could reduce the reliability of the wing size analyzes of individuals.

In our study, only females of the species *P. nimpha* were analyzed because of the insufficient material representing males of this species. Abbasi (2009) reports on the dimorphism of the front pairs of wings between the sexes in the genus *Polistes*. Also Szentgyörgyi et al. (2017) report on differences in the size and shape of wings in both sexes of *Osmia bicornis* (Hymenoptera) developing in an environment contaminated with heavy metals. In this case, the asymmetry of shape and size was smaller in females than in males; therefore, future research should also focus on male *P. nimpha*.

The research carried out so far indicates the existence of a relationship between the symmetry of features of bilateral insects and the basic aspects of their life f.e. sexual selection, mating success (McLachlan and Cant 1995, Pavković-Lučić and Kekić 2011), susceptibility to disease or predatory attack (Møller 1996).

We can presume that features of the wings, including bilateral symmetry, are crucially important in flight performance, which in turn is of main importance in the life of the colony.

Based on the results obtained by us, it can be assumed that the wings of *P. nimpha* females may become a useful object in studying the impact of environmental stress of heavy metal pollution on the symmetry of their wings. It should be remembered that these are preliminary studies, and the results obtained by us require confirmation of this effect by subsequent observations in relation to the above-mentioned species.
